# Impact of Human Umbilical Cord Blood Mononuclear Cells on Gentamicin-Induced Renal Injury and Genotoxicity in Rats

**DOI:** 10.3389/fmed.2021.689691

**Published:** 2021-08-19

**Authors:** Ali H. Abu Almaaty, Reham A. Elmasry, Mayada S. Farrag, Fayez Althobaiti, Adil Aldhahrani, Eman Fayad, Mona A. Hussain

**Affiliations:** ^1^Department of Zoology, Faculty of Science, Port Said University, Port Said, Egypt; ^2^Department of Pathology, Faculty of Medicine, Port Said University, Port Said, Egypt; ^3^Department of Biotechnology, Faculty of Sciences, Taif University, Taif, Saudi Arabia; ^4^Clinical Laboratory Sciences Department, Turabah University Faculty, Taif University, Taif, Saudi Arabia; ^5^Department of Physiology, Faculty of Medicine, Port Said University, Port Said, Egypt

**Keywords:** Gentamicin, nephrotoxicity, genotoxicity, cord blood, stem cell, KIM-1, NGAL, SRY

## Abstract

**Background:** Acute kidney injury (AKI), also known as acute renal failure (ARF), has received considerable critical attention in recent years. Gentamicin (GM) is an antibiotic whose prolonged use results in AKI with a high mortality rate.

**Methods:** Fifty adult female albino rats weighing 150–200 g were used. The animals were divided into five groups: the first group was the normal healthy control one, the second group received only 1 × 10^6^ HUCB mononuclear cells (MNCs)/rat by intravenous (iv) injection, the third diseased group was given GM 100 mg/kg for 10 consecutive days by intraperitoneal injections, the fourth preventive group received 1 × 10^6^ HUCB MNCs/rat by iv injection 24 h before gentamicin treatment, and the fifth treated group received 1 × 10^6^ HUCB MNCs/rat by iv injection 24 h after gentamicin treatment. After 1 week of treatment, blood samples were collected, and kidneys were removed for histopathological examination.

**Results:** Rats treated with HUCB MNCs in the treated group had a significant decrease in renal damage, low levels of biomarkers for nephrotoxicities such as serum creatinine and blood urea nitrogen, and low chromosomal aberrations compared to the diseased third group. The gene expression of KIM-1 and NGAL was decreased in response to HUCB treatment.

**Conclusions:** HUCB MNCs have a curative effect against AKI and gentamicin-induced genotoxicity owing to their regenerative property.

## Introduction

Acute kidney injury (AKI) is characterized as a sudden loss of renal function (within 48 h) caused by an increase in serum creatinine of ≥0.3 mg/dl (26.4 mol/l), a percentage increase in serum creatinine of more than or equal to 50% (1.5-fold from baseline), or a decrease in urine production (documented oliguria of <0.5 ml/kg/h for more than 6 h) or a combination of these factors (1–3).

In recent years, AKI has attracted considerable attention, both scholarly and popular. AKI affects more than 13 million people every year, with a prevalence of 21.6% in adults and 33.7% in children during a single hospitalization episode (4, 5). AKI still has a high mortality rate of 1.7 million per year, with 23.9% in adults and 13.8% in children, and high morbidity and cost (4, 5). AKI costs at least $5 billion in healthcare costs in the United States, while it consumes 1% of the National Health Service budget in England (6). AKI is most often observed in elderly patients and intensive care units in the developed world; however, adults and women are more commonly affected in developing countries (7, 8).

Some medications have multiple effects on renal function (9–11). Drugs are responsible for ~20% of nephrotoxicity (12). In the large community of AKI-associated medications, antibiotics and other antimicrobials are well-known contributors to structural and functional renal dysfunction (13). Gentamicin (GM) is an aminoglycoside antibiotic that treats life-threatening infections and prevents bacteria from producing the protein that usually kills them, especially those caused by gram-negative organisms (14–17). However, it is only used for a brief period due to its high risk (18). The occurrence of GM-induced AKI varies between 2 and 55% of patients. Furthermore, up to 30% of patients given GM for more than 7 days show symptoms of renal dysfunction. Several attempts to prevent GM-induced AKI have failed (19).

Genotoxicity refers to DNA or chromosome damage, leading to gene mutations, chromosome splits, and rearrangements (20). For example, it has been found that GM had a genotoxic effect on bone marrow cells in mice, as demonstrated by an increase in the number of aberrant cells and structural chromosomal aberrations (21). Further, GM-mediated genotoxicity was shown in a pig kidney cell line in an *in vitro* study (22).

Umbilical cord blood (UCB) is the blood that remains in the placenta and attached umbilical cord after childbirth (23). The high concentration of stem/progenitor hematopoietic cells in UCB makes it useful for treating hematopoietic and genetic disorders (23). The use of stem cells to treat complicated conditions like AKI has received increased attention (24). It has been demonstrated that mesenchymal stem cells (MSCs) may help treat AKI (25). Furthermore, stem cell transplantation has been proposed to treat glycerol-induced renal toxicity, which is associated with critical histopathological changes in renal tissue and enhanced kidney function tests (26). The kidney injury molecule-1 (KIM-1) and neutrophil gelatinase-associated lipocalin (NGAL) biomarkers were used to classify renal injury earlier and more specifically due to the low sensitivity and specificity of serum creatinine (SCr) and blood urea nitrogen (BUN) (27). Kidney injury molecule-1 (Kim-1 in rodents, KIM-1 in humans) (28) is a type 1 transmembrane protein, usually absent but activated when the proximal tubular apical membrane is damaged (26, 27). NGAL is a protein that was first identified in human neutrophils and has since been found in immune cells, various tissues, and organs such as the trachea, lung, intestine, liver, colon, and kidney (29).

The current study set out to investigate whether mononuclear stem cells from human UCB could be a beneficial therapeutic agent for AKI model and genotoxicity in rats, especially GM-induced bone marrow genotoxicity.

We hypothesized that SCs would have a therapeutic effect on injured kidneys in rats treated with HUCB MNCs after GM injections and that SCs may prevent harm in rats treated with SCs before GM.

## Materials and Methods

### Induction of GM-Induced Renal Toxicity

GM (Memphis Co. for Pharm. & Chem. Ind., Cairo, A.R.E.) was injected intraperitoneally at a dose of 100 mg/kg for 10 consecutive days (17) in the diseased and treated groups before HUCB MNC transplantation and in the prevention group after HUCB MNC transplantation. According to a pilot study, the dose was determined.

### Experimental Animals

This study was carried out following the recommendations for the care of laboratory animals published by the US National Institute of Health (NIH Publication No. 85.23, revised 1985). Moreover, it has been reviewed and approved by the animal care and use according to the Zoology Department, Faculty of Science, Port Said University. Fifty Sprague Dawley albino female rats, weighing 150–200 g, were housed in standard rat plastic cages for 2 weeks before being exposed to a 12-h light/12-h dark cycle at a controlled temperature of 23 ± 2°C. Rats were given food (pellets) and water.

**Figure 1:**
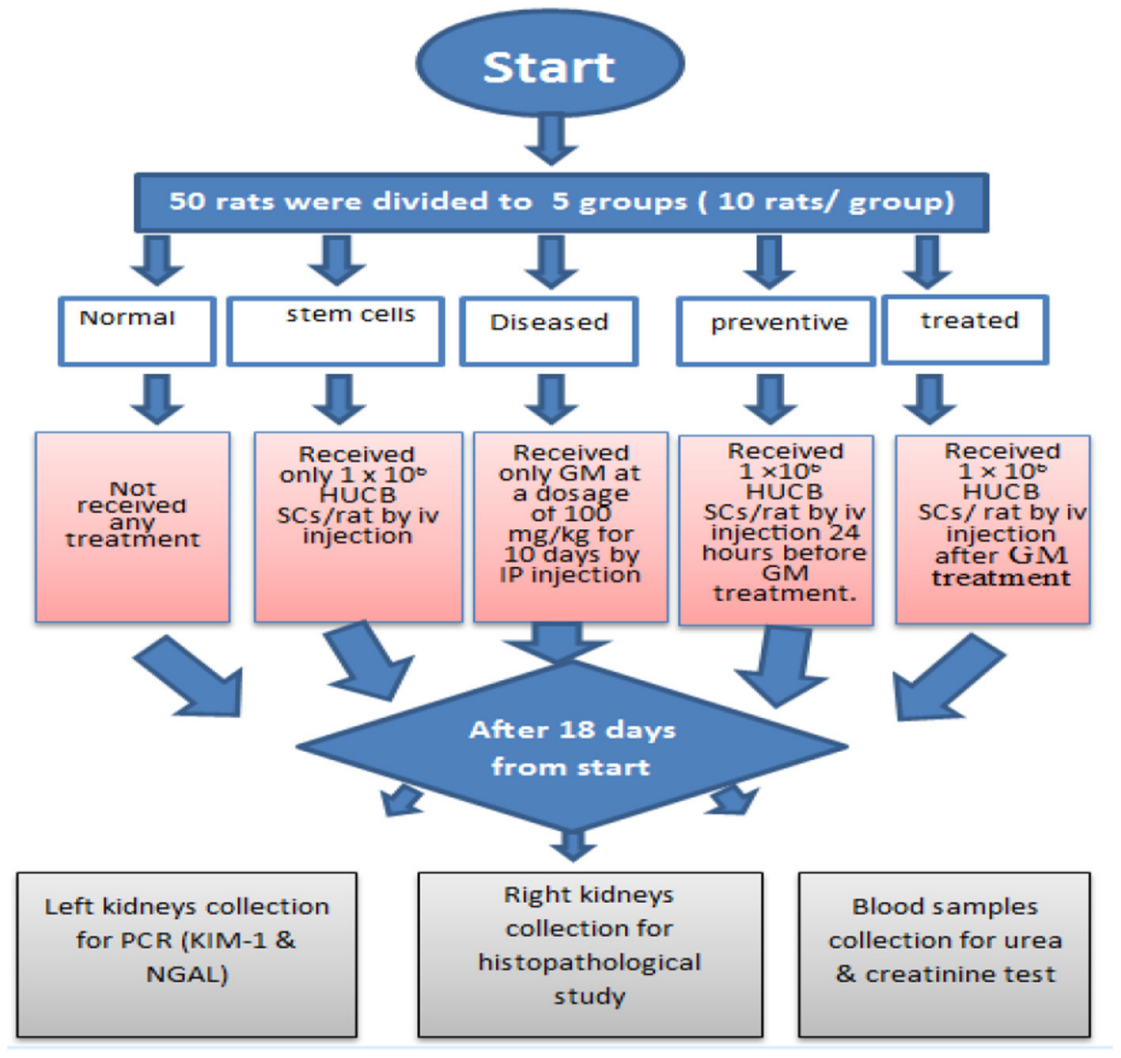
Consort-flow diagram for the experiment.

The rats were randomly divided into five groups (see Figure 1):

1. **The first group (Normal)** received no treatment.
2. **The second group (stem cells)** received only 1× 10^6^ HUCB MNCs/rat by iv injection.
3. **The third group (Diseased)** received only saline GM at a dosage of 100 mg/kg for 10 consecutive days by intraperitoneal injections.
4. **The fourth group (preventive)** received 1× 10^6^ HUCB MNCs/rat by iv injection 24 h before saline GM treatment.
5. **The fifth group (treated)** received 1 × 10^6^ HUCB MNCs /rat by iv injection 24 h after saline GM treatment.

### HUCB Cell Isolation and Transplantation

Three 50-ml blood samples were collected using sterile collection tubes (50 ml) containing 5 ml citrate phosphate dextrose adenine-1 (CPDA-1) as an anticoagulant immediately after cesarean maternal donor deliveries. Mononuclear stem cells were isolated by Ficoll-Hypaque density gradient centrifugation (Biochrom GmbH, Berlin, Germany) (30). A volume of 0.2 ml phosphate-buffered saline solution was added to the HUCB MNC pellet for injection of 1 × 10^6^ cells/rat by iv injection into the lateral tail vein using a Hamilton syringe in the control stem cell group, the treated group 24 h after GM treatment, and the preventive group before GM treatment (31).

### Blood Chemistry

The rats fasted overnight before the autopsy after 1 week of treatment. The retro-orbital blood samples were collected into serum separator tubes and then centrifuged at 1,500 × *g* for 10 min at 4°C. Isolated serum samples were stored at −80°C until use. Serum SCr and BUN were measured using a standard clinical chemistry analyzer (RIELE Photometer 5010 V5+ semi-automated clinical chemistry analyzer, Robert Riele GmbH & Co. KG, Berlin, Germany).

### Histopathological Studies

Following 48 h of fixation in 4% paraformaldehyde, the right kidneys were rinsed in tap water, dehydrated in a graded alcohol sequence, embedded in paraffin, and sectioned into 5-μm-thick sections. Hematoxylin–eosin staining was performed on paraffin sections. Histopathological damage indices including mononuclear cell infiltration, tubular degeneration, tubular necrosis, and tubular casts were observed using a light microscope. These changes were evaluated and graded as follows: zero for no detectable lesion, 1 for mild changes, 2 for moderate changes, and 3 for severe changes (32).

### Detection of Chromosomal Abnormalities

After 1 week of treatment, rats were euthanized, bone marrow from the femur was excised, and air-dried metaphase preparations were performed using the technique of Tijo and Whang (33). The slides were subsequently stained with 10% Giemsa dye (stock solution: 3.8 g Giemsa, 250 ml glycerol, 250 ml methanol) and 50 metaphases per group were assessed. Hence, 250 metaphases were evaluated for various chromosomal aberrations such as polyploidy, hypoploidy, deletion, fragments, chromatid breaks, chromosome breaks, ring chromosome, and chromatid gap in each experiment. The slides were photographed under a light microscope (Leica, Wetzlar, Germany).

### Gene Expression for Kim-1, NGAL, and SRY

According to the manufacturer′s instructions, total RNA was extracted from the left kidney of three rats/group using the Qiagen tissue extraction kit (Qiagen, Germantown, MD, USA). The purity (*A*260/*A*280 ratio) and RNA concentration were then obtained using spectrophotometry (dual-wavelength Beckman spectrophotometer, Beckman Foundation, Irvine, CA, USA). The total RNA (0.5–2 μg) was used for cDNA conversion using a high-capacity cDNA reverse transcription kit (Fermentas, Waltham, MA, USA). Kim-1, NGAL, and SRY gene expression was evaluated by real-time PCR through 40 cycles using a commercial SYBR Green Master Mix (SensiFAST SYBR, Bioline, London, UK). Five microliters of cDNA template, 1 μl of each forward and reverse primer, 12.5 μl of a SYBR Green mix, and 5.5 μl RNAse-free water were combined to make a total volume of 25 μl that was introduced to thermal cycler instrument (Thermo Scientific, Waltham, MA, USA). The mixture was incubated in the programmed thermal cycler for 1 h at 37°C, followed by inactivation of enzymes at 95°C for 10 min, and finally cooled at 4°C. RNA was then changed into cDNA that was stored at −20°C. In this study, gene expression was compared to that of the normal healthy group. The primers used are shown in Table 1. The relative quantitation (RQ) was calculated according to Applied Biosystems Software using the following equation

$$\begin{array}{lll} \Delta \text{Ct} & = & \text{Ct gene test}-\text{Ct endogenous control}\\ \Delta \Delta \text{Ct} & = & \Delta \text{Ct sample}1-\Delta \text{Ct calibrator}\\ \text{RQ} & = & \text{Relative quantification}=2^{-\Delta \Delta \text{Ct}} \end{array}$$

The RQ is the fold change compared to the calibrator (untreated sample).

**Table 1:** The primer sequences for real-time PCR assay.

**Gene**	**Sequences (5** ^**′**^ **-3** ^**′**^ **)**	**Product length**	**Accession number**
Kim-1	Forward primer: 5′-AACGCAGCGATTGTGCATCC-3′	697	NM_173149.2
	Reverse primer: 5′-GTACACTCACCATGGTAACC-3		
NGAL	Forward primer 5′-GATGAACTGAAGGAGCGATTC-3′	83	NM_130741.1
	Reverse primer 5′-TCGGTGGGAACAGAGAAAAC-3		
SRY	Forward primer 5′-CATCGAAGGGTTAAAGTGCCA-3′	104	XM_008773686.3
	Reverse primer 5′-ATAGTGTGTAGGTTGTTGTCC-3′		

### Statistical Analysis

Data were expressed as mean ± SD and analyzed using the Statistical Package for Social Science (SPSS) 22 software (USA). Parametric data were analyzed by ANOVA followed by Bonferroni *post hoc* test and non-parametric data were analyzed by Chi-Square. The exact Fisher test was used for assessment of the significance of histopathological changes. The *p-*value < 0.05 was considered significant.

## Results

### Kidney Function Test Results

**Figure 2:**
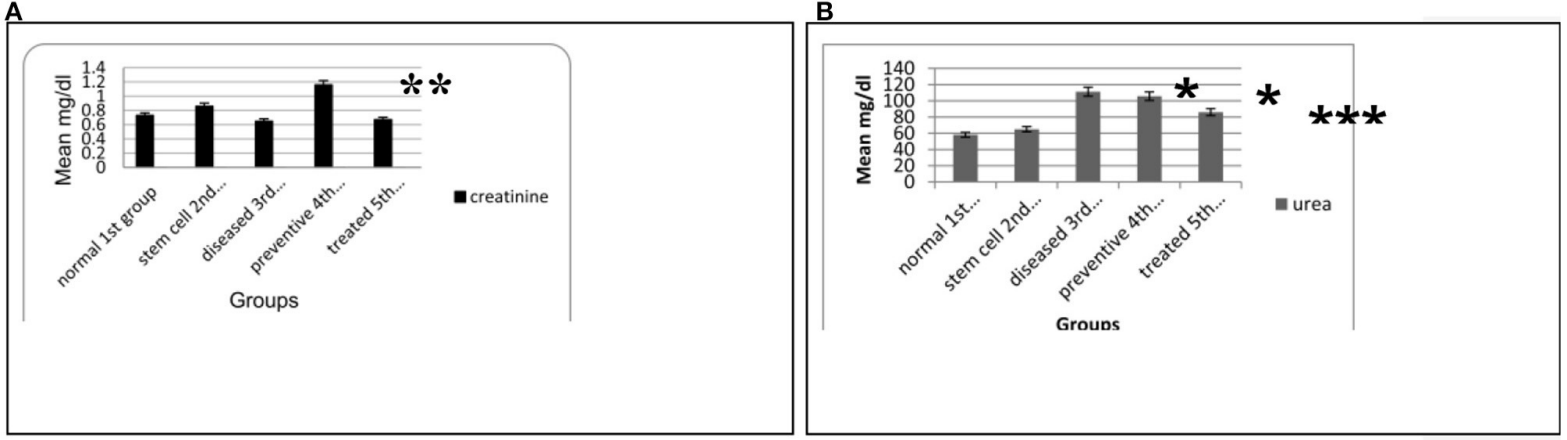
**(A)** Means of SCr level in the blood serum for the different animal experimental groups. **(B)** Means of BUN level in the blood serum for the various animal experimental groups. Each data are the mean ± S.D using ANOVA, *p* < 0.05. *Significant compared to other groups, *p* < 0.05. **Significant compared to the normal and stem cell groups, *p* < 0.01. ***Significant compared to the diseased group, *p* < 0.05.

The mean traditional biomarkers for nephrotoxicity Scr and BUN in blood serum showed a marked increase in SCr in the fourth preventive group compared to other groups (*p* = 0.001 and 0.043). In contrast, serum urea levels increased significantly in the preventive fourth group compared to the normal first and stem cell second groups (*p*= 0.001). Moreover, a significant increase in serum urea level was found in the diseased third group compared to the stem cell second group and normal first group (*p* = 0.001). Further, a substantial decrease in the serum urea level was observed in the treated fifth group compared to the diseased third group (*p* = 0.04) (see Figure 2).

### Histopathological Results

**Figure 3:**
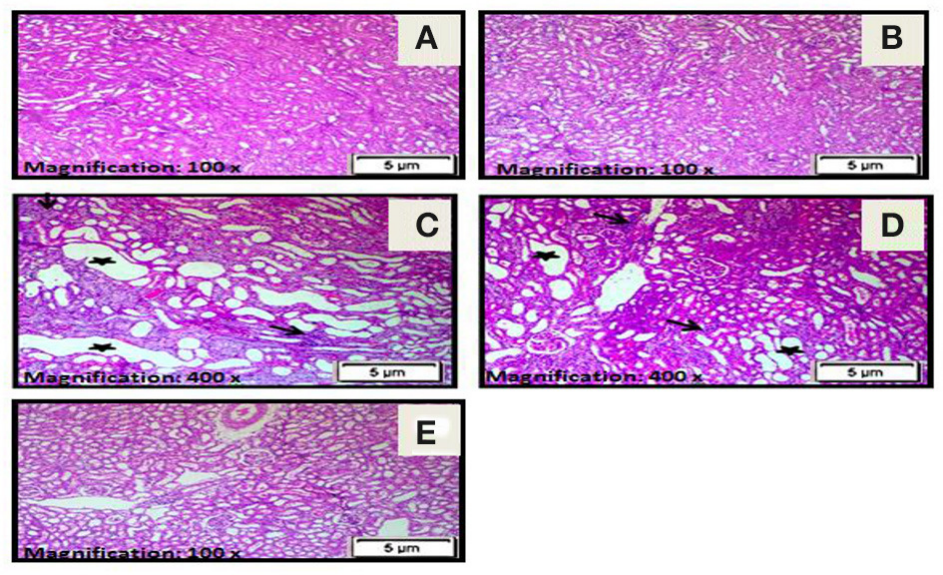
**(A)** A photomicrograph of the section from the cortex of the rats in the control normal first group revealed a normal histology of kidney tissue. **(B)** A photomicrograph of the section from the cortex of rats in the stem cell second group showed a normal histology of the kidney cortex. **(C)** Photomicrographs of sections from the cortex of rats in the diseased third group demonstrated marked tubular necrosis with markedly dilated irregular proximal tubules with attenuation or loss of lining epithelial cells (star) and interstitial mononuclear inflammatory cell infiltration (arrow). **(D)** Photomicrographs of sections from the cortex of rats in the preventive fourth group showed moderate tubular necrosis with dilated irregular proximal tubules and attenuation or loss of lining epithelial cells (star) together with interstitial mononuclear inflammatory cells infiltration (arrow). **(E)** A photomicrograph of the section from the cortex in the treated fifth group rats showed resolution of all histopathological alterations with tubular regeneration and resolution of interstitial inflammatory infiltrate (H&E × 100).

H&E staining revealed apparent pathological lesions in the kidneys of the diseased third and preventive fourth groups compared to the treated fifth group, including mononuclear inflammatory cell infiltration, tubular epithelial cell loss and necrosis, and tubular casts. In contrast, the normal first group and stem cell second group had normal histology of the kidney tissue (see Table 2; Figure 3).

**Table 2:** Kidney histopathological changes (mononuclear cell infiltration, tubular degeneration, tubular cell necrosis, and tubular casts) in the study groups.

	**Mononuclear cells infiltration**				**Tubular degeneration**				**Tubular cell necrosis**				**Tubular casts**			
	**0**	**1**	**2**	**3**	**0**	**1**	**2**	**3**	**0**	**1**	**2**	**3**	**0**	**1**	**2**	**3**
Normal	2	2	0	0	3	1	0	0	4	0	0	0	3	1	0	0
Stem cell	4	0	0	0	2	2	0	0	4	0	0	0	2	2	0	0
Diseased	0	0	1	3	0	0	4	0	0	0	1	3	0	1	1	2
Preventive	0	0	3	1	0	0	4	0	0	0	4	0	0	4	0	0
Treated	2	2	0	0	1	3	0	0	3	1	0	0	2	2	0	0
*P*-value	0.003^**^				0.003^**^				0.000^**^				0.064			

*Semiquantitative scoring system of histopathological changes in the study groups (four rats/group)*.

** *Highly significant difference among the study groups, p < 0.05*.

### Cytogenetic Results

**Figure 4:**
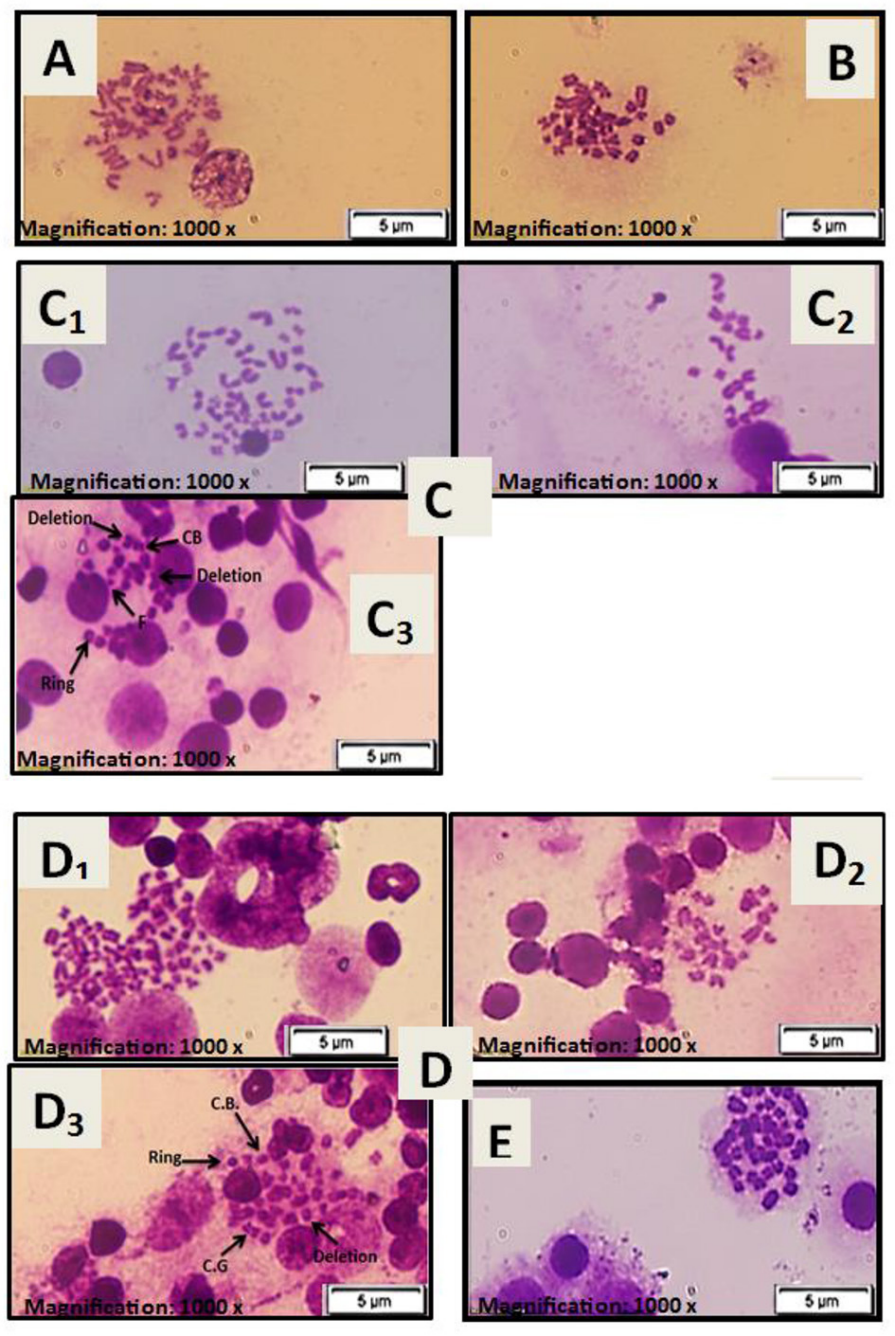
**(A)** Metaphase spread of female rat bone marrow cells for the normal rat. **(B)** Metaphase spread of female rat bone marrow cells for rat injected with stem cells only at a dose of 1 × 10^6^ cells/rat. **(C)** Metaphase spread of female rat bone marrow cells for the diseased group injected with only GM at a dose of 100 mg/kg: (C1) chromosomal polyploidy, (C2) chromosomal hypoploidy, and (C3) chromosomal aberrations: chromosomal break (CB), deletion, chromosomal ring (Ring), and fragments (F). **(D)** Metaphase spread of female rat bone marrow cells for the preventive group treated with stem cells at a dose of 1× 10^6^ cells/rat before being injected with GM at 100 mg/kg: (D1) chromosomal polyploidy, (D2) chromosomal hypoploidy, and (D3) chromosomal aberrations: chromosomal break (CB), deletion, chromosomal ring (Ring), and chromatid gap (CG). **(E)** Metaphase spread of female rat bone marrow cells for the treated group injected with GM at a dose of 100 mg/kg and then treated with stem cells at a dose of 1× 10^6^ cells/rat.

There were normal chromosomes with a basal level of aberrations in the normal first and stem cell second groups (see Figures 4A,B). However, severe chromosomal abnormalities, such as polyploidy, hypoploidy, deletion, fragments, chromatid breaks, chromosomal break, ring chromosome, and chromatid gap, were found in the diseased third and preventive fourth groups (see Figures 4C,D). After treatment with HUCB MNCs in the fifth group, there was a low frequency with a significant record for chromosomal aberrations (see Figure 4E).

### Gene Expression Results

**Figure 5:**
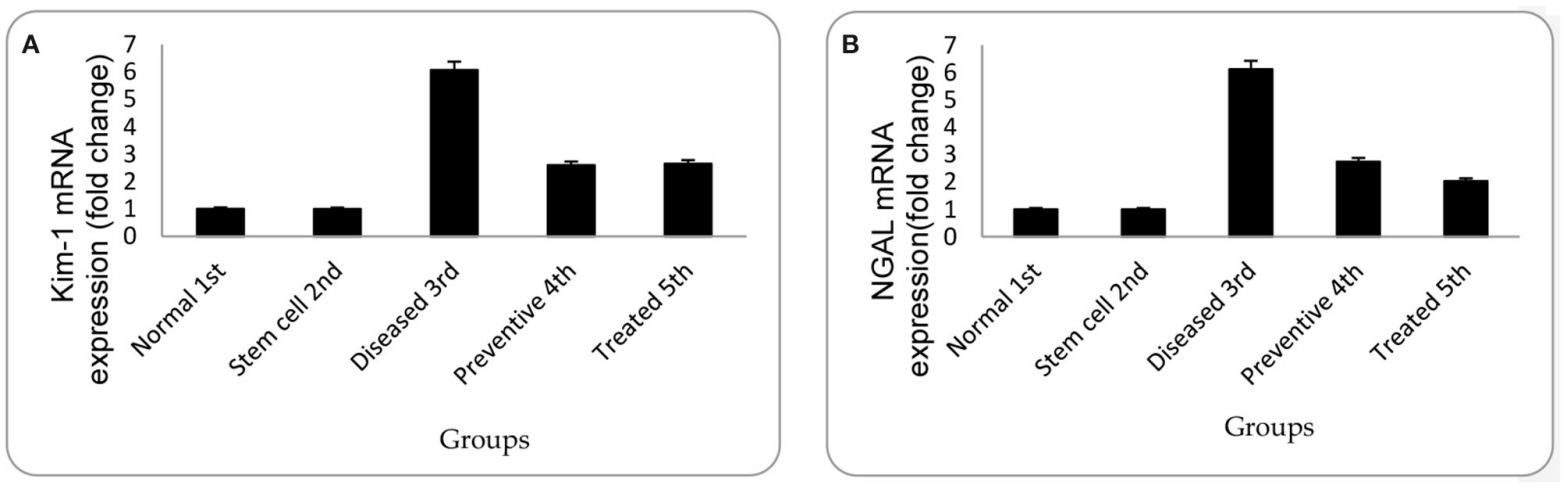
**(A)** Relative gene expression of Kim-1 for mean of experimental groups with their standard deviation. **(B)** Relative gene expression of NGAL for the mean of experimental groups with their standard deviation. Each data are presented as mean fold change ± SD using ANOVA, *p* < 0.05.

**Figure 6:**
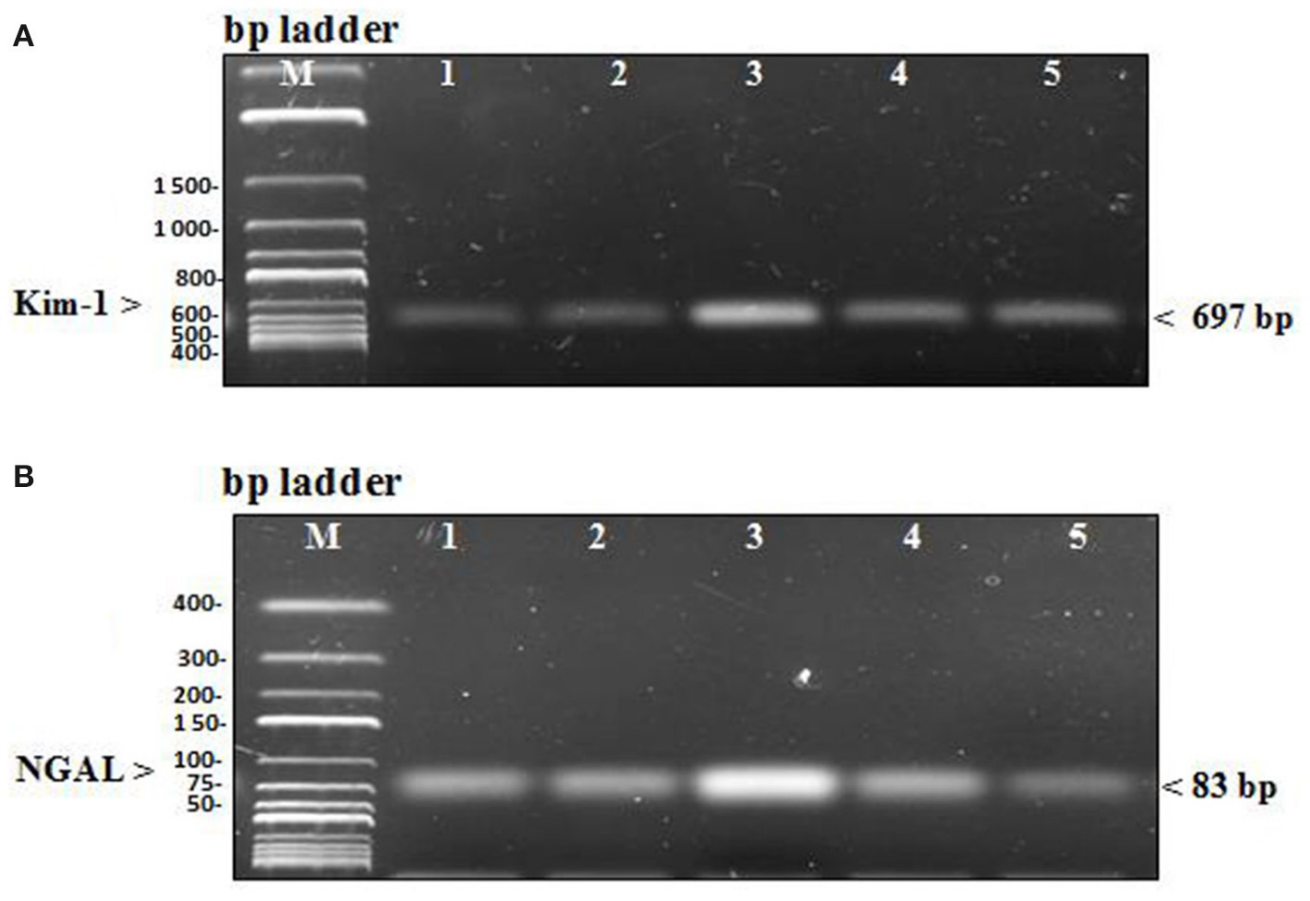
(**A**) PCR products of renal injury mRNA biomarker Kim-1 were separated from kidney tissue of rats on agarose gel electrophoresis (**B**) PCR products of renal injury mRNA biomarker NGAL were separated from kidney tissue of rats on agarose gel electrophoresis. In each figure, Lane M: DNA molecular mass marker, lane 1 and 2: PCR products of Kim & NGAL gene expression in the control group that received no treatment and the stem cell group that received only HUCB MNCs, lane 3: PCR products of Kim & NGAL gene expression in the diseased group receiving GM only, lane 4 and 5: PCR products of Kim & NGAL gene expression in the preventive group that received HUCB MNCs before injecting with GM and the treated group that obtained GM injections followed by HUCB MNCs. As demonstrated, renal injury biomarker expression was significantly higher in the diseased group than in the other studied groups.

**Figure 7:**
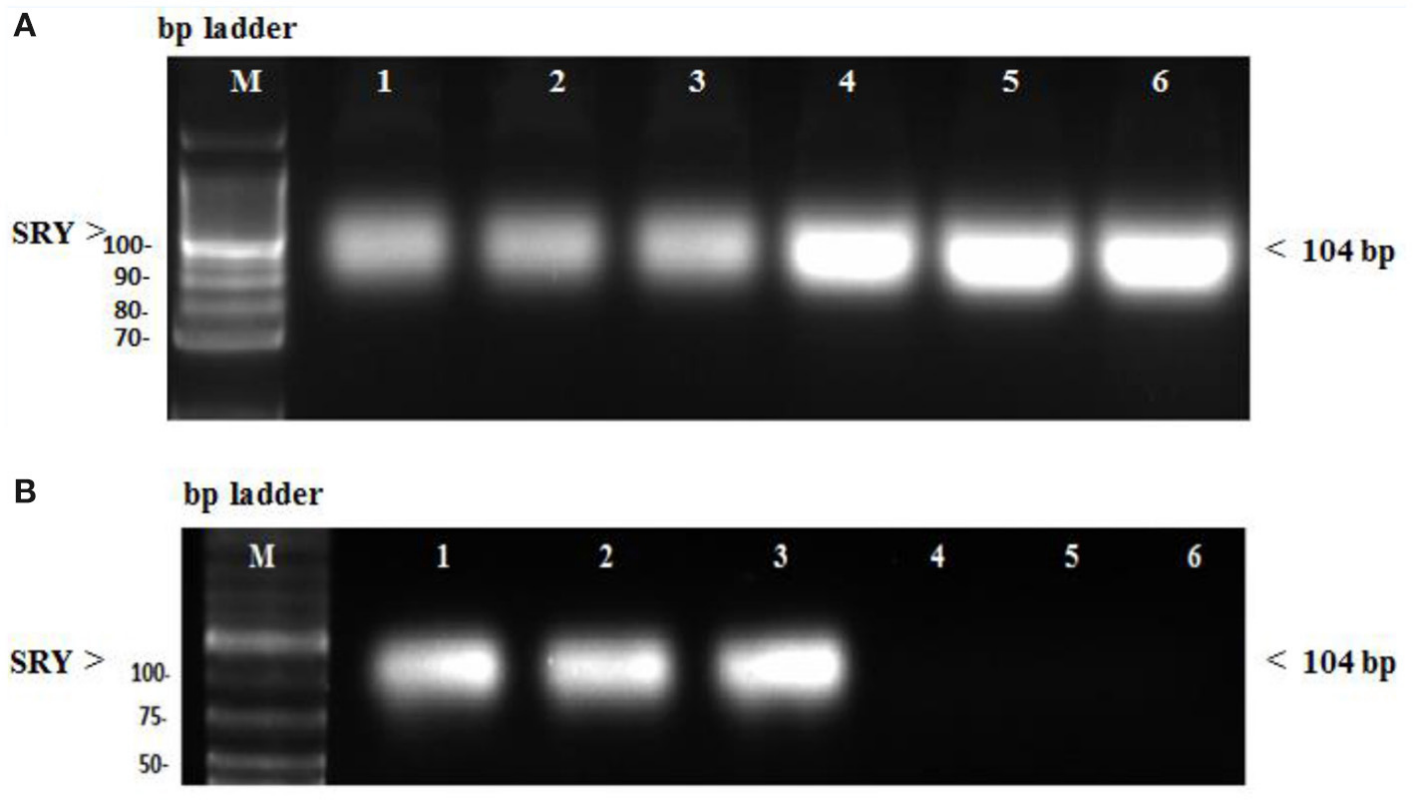
**(A)** SRY gene expression in female kidney tissue rats evaluated by conventional PCR. Lane M: DNA molecular mass marker. Lane 1–3: sample injected with only stem cells (second group). Lane 4–6: sample treated with stem cells before being injected with GM (preventive fourth group). **(B)** SRY gene expression in female kidney tissue rats evaluated by conventional PCR. Lane 1–3: sample injected with GM, then treated with stem cells (treated fifth group). Lane 4–6: sample did not receive any treatment (normal control first group).

In the diseased third group, Kim-1 and NGAL expressions in kidney tissues were significantly higher than in the other groups (*p* = 0.001 vs. control, stem cell, and treated groups, *p* = 0.003 vs. preventive group) (see Figure 5 and Figure 6). Human SRY gene was detected in the kidney tissues of female rats of all groups injected with SCs (see Figure 7).

## Discussion

### Correlation Between Nephrotoxicity and Genotoxicity

There was a strong significant correlation between the renal histopathological findings and the KIM-1 & NGAL expression in the study groups (*p* = 0.00) (Table 3). The current study uses an aminoglycoside antibiotic (GM) as a compound model to investigate whether adult stem cells from cord blood may treat AKI and genotoxicity in rats. Unfortunately, AKI is associated with a high death rate, requiring early non-invasive and simple marker production for speedy AKI determination (15). It has been reported that SCr and BUN are potentially linked to renal injury following GM-induced AKI (15). HUCB has emerged as a source of cells with therapeutic potential in many medical fields (34). Previous studies have shown that HUCB MNCs are useful for treating hematopoietic and genetic disorders (23), diabetes (30), AKI caused by glycerol (26) and diabetic nephropathy (35).

**Table 3:** Pearson correlation (*r*) between renal histopathological findings and genetic expression of KIM-1 and NGAL in the study groups.

**Histopathological changes**	**Tubular cell degradation (TD)**	**Mononuclear infiltration (MI)**	**Tubular casts (TC)**	**Tubular cell necrosis (TN)**
KIM	0.671^**^	0.779^**^	0.835^**^	0.862^**^
NGAL	0.698^**^	0.770^**^	0.855^**^	0.888^**^

** *p < 0.01*.

*There was a strong correlation among the renal histopathological findings and the genetic expression of KIM-1 and NGAL expression in the study groups*.

We found that a loading dose of 1 × 10^6^ HUCB MNCs/rat could ameliorate GM-induced AKI successfully. In addition, improvements in the kidney function test in the treated community were significant. However, blood urea and serum creatinine have low sensitivity and specificity because SCr and BUN are affected by many non-renal factors that are independent of kidney injury or kidney function like muscle mass, infection, age, production and renal tubular handling, sex, volume of distribution, race, and nutritional status (36, 37). Moreover, actual alterations may take several days to manifest in SCr (38).

Due to the presence of megalin and cubilin, which are membrane endocytic proteins, GM deposits in the epithelial cells of proximal renal tubules, altering phospholipid and lipid metabolism and lysosomal aggregation (39), as well as changing the function of epithelial cells of proximal renal tubules *via* alteration of extracellular calcium-sensing receptors, leading to necrosis and cell death (9, 40, 41). However, GM-induced renal ischemia leads to induction of inducible nitric oxide synthase that causes mitochondrial oxidative stress and inhibition of ATP production in glomerular endothelial cells causing inflammation of glomeruli. GM-induced AKI is a multifaceted phenomenon in which a high dose of GM causes free-radical generation and oxidative stress. Besides, GM stimulates the mitochondrial respiratory chain for generating free radicals. Previous studies documented that GM therapy led to a significant reduction in the activity of glutathione, superoxide dismutase, and other endogenous antioxidant capacities, which may have contributed to GM-induced AKI (9, 41).

The results showed that rats treated with HUCB MNCs after GM injections revealed a significant improvement in renal tissue histopathological changes correlated with renal tubular injury score and H&E staining. Many studies have shown that the beneficial effect of SCs might be mediated by downregulating proinflammatory cytokines (interleukin IL-6, interleukin IL-12, tumor necrosis factor TNFα, interferon-gamma IFN-γ) and upregulating anti-inflammatory cytokines (interleukin IL-10) (9). Furthermore, humoral effects cause HUCB stem cells to reduce natural killer T cell infiltration and increase regulatory T cells (42).

The existence of metaphase spread defects, as evaluated by genotoxicity tests, indicates that GM has hazardous effects on bone marrow chromosomes. Severe chromosomal aberrations, such as polyploidy, hyperploid, deletion, fragments, chromatid breaks, chromosome breaks, ring chromosome, and chromatid gap, were shown in the diseased and preventive groups. After injection with GM, the use of HUCB MNCs showed a reduction in chromosomal abnormalities and gene biomarkers (Kim-1, NGAL) in the treated group. KIM-1 is usually absent but activated when the proximal tubular apical membrane is damaged due to tubular necrosis with a consequent decrease in the number of functioning nephrons (26). Hence, we suggested that HUCB-MNCs could restore the normal expression of Kim-1 level by minimizing the observed renal damage. It has been proposed that inflammation plays a role in the pathogenesis of GM-induced nephrotoxicity (43).

On the other hand, several studies found that the NGAL level is elevated with inflammation. Thus, the anti-inflammatory effect of HUCB MNCs may be responsible for restoring the regular expression of NGAL despite GM treatment (35, 44, 45). Kim-1 and NGAL are more specific for predicting AKI since they are tested in different types of AKI and larger clinical studies, allowing earlier detection of kidney injury before an increase in SCr and/or BUN and before the initiation of renal proximal tubules damages. Accordingly, we can monitor the effects of an intervention or treatment (46).

The results showed a strong significant correlation between the renal histopathological findings and the genetic expression of KIM-1 and NGAL expression in the study groups. Many studies suggested the role of reactive oxygen metabolites in GM toxicity (47). GM was found to enhance the generation of superoxide anions and hydroxyl radicals from renal cortical mitochondria (47). These oxygen free radicals play an important role in the pathogenesis of nephrotoxicity by GM that is indicated by an increased number of aberrant cells and some kinds of structural chromosomal aberrations (48). Furthermore, many authors approved the straight correlation between genotoxicity and chromosomal instability induced by many agents with the oxidative stress parameters (12, 41, 49).

Interestingly, many studies evaluated the presence of SCs in renal tissues by using the chromosome Y localization strategy (50, 51). We detected the human SRY gene in kidney tissues of all groups injected with SCs. These results match those observed in previous studies, demonstrating that the injected SCs could be detected over a lengthy period (52, 53). Many animal and human studies have shown the role of stem cells in kidney repair and regeneration. Thus, stem cell-based therapy appears to be a promising new candidate for AKI management (54). SCs' regenerative effect was assumed to be due to their paracrine/endocrine activity, which includes releasing bioactive factors that act on injured renal cells and producing proangiogenic, antiapoptotic, antioxidant, and immunomodulatory effects (55, 56).

The administration of HUCB MNCs after the last injection of GM may have been more effective in preventing the progression of renal injury. The most severe tubular damage was found histologically after 10 days of G administration, where 75–90% of the outer cortical tubules were necrotic (57). Blood and kidney samples were obtained after 24 h of HUCB MNC administration. Previous experiments have linked the timing and route of SCs administration to the protective effect against AKI (58).

Some animal models have been used to investigate possible pathways for HUCB MNCs' protective properties (26, 35, 43, 58–60). Most SCs were gradually transferred to injured sites or the liver, spleen, kidney, and bone marrow (61, 62) to recruit SCs to repair the damage caused by release factors (63). HUCB MNCs were found to reduce renal impairment in the early stages of ischemia–reperfusion injury through humoral effects and production of vascular endothelial growth factor (31, 42, 64, 65). SCs have been shown to have low immunogenicity due to the low number of major histocompatibility complex (MHC) molecules present on their surface. Besides, chemokines that alter the immune response and promote tolerance of the new tissue have been secreted, enabling allogeneic treatment to be carried out with a low probability of rejection (66). However, in rats treated with HUCB MNCs before injection with GM, the improvement in AKI complications was relatively weak, despite the presence of the SRY gene in their kidney tissues, suggesting that SCs were unable to prevent GM-induced injury before usage. However, tissue injury could be reduced if SCs were transplanted during the early stages of AKI (56).

The study's limitations included the lack of measurement of oxidative stress marker and inflammatory markers.

## Conclusions

Our hypothesis of the study was validated in terms of the curative impact of HUCB MNC transplantation on nephrotoxicity, as SCs could improve the injured kidney after GM injection, but HUCB MNC transplantation before GM injections could not protect the kidney from injury. After injection with GM, the use of HUCB MNCs showed a significant reduction of chromosomal abnormalities and biomarker level in the treated group. Also, a significant improvement in renal tissue histopathological changes correlated with renal tubular injury was found. Besides, the marked SCr and BUN improved levels proved the repairing role of stem cells in the kidney. Different preventive modalities for GM-induced AKI and genotoxicity require further research.

## Data Availability Statement

Requests to access the datasets should be directed to hhhh_fayed@yahoo.co.uk.

## Ethics Statement

This study was carried out following the recommendations for the care of laboratory animals published by the US National Institute of Health (NIH Publication No. 85.23, revised 1985). Moreover, it has been reviewed and approved by the animal care and use according to Zoology Department, Faculty of Science, Port Said University.

## Author Contributions

AHA, MH, and RE: conceptualization and methodology. MF, MH, and RE: formal analysis. AHA, MH, EF, and RE: writing—original draft preparation and data curation. AHA, MH, RE, FA, AA, and EF: writing, review, and editing. AHA, MH, EF, MF, and RE: funding acquisition and validation. MH: visualization. AHA and MH: supervision. RE, FA, AA, and EF: project administration. All authors contributed to the article and approved the submitted version.

## Conflict of Interest

The authors declare that the research was conducted in the absence of any commercial or financial relationships that could be construed as a potential conflict of interest.

## Publisher's Note

All claims expressed in this article are solely those of the authors and do not necessarily represent those of their affiliated organizations, or those of the publisher, the editors and the reviewers. Any product that may be evaluated in this article, or claim that may be made by its manufacturer, is not guaranteed or endorsed by the publisher.
